# MicroRNA-155 acts as an anti-inflammatory factor in orbital fibroblasts from Graves’ orbitopathy by repressing interleukin-2-inducible T-cell kinase

**DOI:** 10.1371/journal.pone.0270416

**Published:** 2022-08-18

**Authors:** Yeon Jeong Choi, Charm Kim, Eun Woo Choi, Seung Hun Lee, Min Kyung Chae, Hyung Oh Jun, Bo-Yeon Kim, Jin Sook Yoon, Sun Young Jang

**Affiliations:** 1 Department of Ophthalmology, Soonchunhyang University Bucheon Hospital, Soonchunhyang University College of Medicine, Bucheon, Republic of Korea; 2 Department of Ophthalmology, Severance Hospital, The Institute of Vision Research, Yonsei University College of Medicine, Seoul, Republic of Korea; 3 Division of Endocrinology and Metabolism, Department of Internal Medicine, Soonchunhyang University Bucheon Hospital, Soonchunhyang University College of Medicine, Bucheon, Republic of Korea; Northwest University, UNITED STATES

## Abstract

To investigate the role of microRNA (miR)-155 in inflammation in an in-vitro model of Graves’ orbitopathy (GO). The expression levels of miR-155 were compared between GO and non-GO orbital tissues. The effects of inflammatory stimulation of interleukin (IL)-1β and tumour necrosis factor alpha (TNF-α) on miR-155 expression on GO and non-GO orbital fibroblasts (OFs) were investigated. The effects of miR-155 mimics and inhibitors of inflammatory proteins and IL-2-inducible T-cell kinase (ITK) expression were examined, along with those related to the knockdown of ITK with siITK transfection on inflammatory proteins. We also examined how ITK inhibitors affect miR-155 expression in GO and non-GO OFs. The expression levels of miR-155 were higher in GO orbital tissues than in non-GO tissue. The overexpression of miR-155 was induced by IL-1β and TNF-α in OFs from GO and non-GO patients. IL-1β-induced IL-6 (ICAM1) protein production was significantly reduced (increased) by miR-155 mimics and inhibitors. The mRNA and protein levels of ITK were downregulated by overexpressed miR-155 via miR-155 mimics. Knockdown of ITK via siITK transfection induced a decrease in the expression levels of ITK, IL-17, IL-6, IL-1β, and TNF-α protein. The expression of miR-155 was significantly downregulated by treatment with ITK inhibitors and Bruton’s tyrosine kinase (BTK)/ITK dual inhibitors in a time-dependent manner. Our results indicated a potential relationship between miR-155 and ITK in the context of GO OFs. The overexpression of miR-155 repressed ITK expression and relieved inflammation. Thus, miR-155 appears to have anti-inflammatory effects in GO OFs. This discovery provides a new concept for developing GO treatment therapeutics.

## Introduction

Graves’ orbitopathy (GO) is an autoimmune inflammatory disorder of the orbit. Although the exact pathogenesis remains unclear, our knowledge of GO pathophysiology has improved on both the genetic and molecular levels [1]. In recent years, we have reported several novel results regarding the role of microRNA (miR) in GO pathogenesis [2–4]. We found that miR-146a contributes to GO pathogenesis by modulating inflammatory [2] and fibrotic protein [4] expression in GO orbital fibroblasts (OFs). We also discovered that miR-27a and b inhibit adipogenesis in OFs from GO patients, suggesting the possibility that miR-27 is involved in GO pathogenesis.

MiRs are non-coding, short, single-strand RNAs that modulate pathological and physiological processes by inhibiting target gene expression through blockade of protein translation or induction of mRNA degradation **[5]**. Previous studies have suggested that miRs play an important role in the pathophysiology of autoimmunity and proliferation **[6]**. In particular, miR-210 **[7]**, miR-155 **[7]**, miR-27 **[3]**, and miR-146a **[2,7]** likely influence the pathogenesis of Graves’ disease and GO, and thus may have potential as diagnostic markers. MiR-155 is one of the most prominent miRs linked to inflammation **[8]**, which underlies our interest. This is because the main pathomechanism of GO involves three mechanisms: inflammation, adipogenesis, and glycosaminoglycan accumulation **[9–11]**.

It has been demonstrated that miR-155 acts on more than one receptor and may induce the opposite action with respect to inflammation. Li *et al* **[12]** reported that miR-155 acts as an anti-inflammatory factor in atherosclerosis-associated foam cell formation by repressing calcium-regulated heat-stable protein 1 (CARHSP1). In contrary, MiR-155 expression can be induced by the Toll-like receptor 4 (TLR4)/nuclear factor kappa-light-chain-enhancer of the activated B-cell (NF-κB) pathway; upregulation thereof has been demonstrated in both myeloid and lymphoid-activated cells **[13]**. MiR-155 promotes inflammation by targeted suppression of the cytokine signaling 1 (SOCS1) and SH2-containing inositol phosphatase 1 (SHIP1) regulators, two important negative regulators in the TLR4/NF-κB pathway **[13]**.

IL-2-inducible T-cell kinase (ITK) is a non-receptor tyrosine kinase expressed in T-cells, natural killer cells and mast cells, which plays a crucial role in regulating the T cell receptor and Fc receptor initiated signal transduction [14]. Previous studies have shown that ITK is involved in the pathogenesis of autoimmune diseases [15]. In the present study, we focused on ITK as a target gene of miR-155 because ITK has a binding site for miR-155. We first investigated the role of miR-155 in inflammation in an in-vitro model of GO. Second, we aimed to investigate the potential relationship between miR-155 and ITK, which is known to be an important mediator of inflammation.

## Methods

### Subjects and cell culture

Orbital fat/connective tissue explants were acquired from 12 GO patients and 10 age- and sex-matched control subjects without a history of GO. Orbital adipose tissues were obtained from GO patients as surgical waste during orbital decompression for exophthalmos. These patients had not been treated with steroid medication or radiation therapy for at least 3 months prior to the study and were of euthyroid status at the time of surgery. Non-GO patients included those who underwent lower lid blepharoplasty without any prior medical history. The clinical characteristics of the patient population of this study are detailed in the Table 1. Written informed consent was obtained from all subjects for study participation. This study was approved by the Institutional Review Board of Soonchunhyang Hospital, Soonchunhyang University College of Medicine (2020-04-006).

**Table 1:** Demographic and clinical data in Graves’ orbitopathy (GO) and control groups.

	GO (n = 12)	Control (n = 10)
Age (years)	48.8 ± 15.0	50.8 ± 12.8
Female, n	8	7
Smoker, n	5	3
Clinical activity score (CAS)	0–1 = 8 2–3 = 4 > 3 = 0	NA
Treatment of GO		NA
Previous radiotherapy.	1	
Systemic glucocorticoid		
Oral, > 6 months prior to surgery	4	
Intravenous methylprednisolone	7	
Glucocorticoids at the time of surgery	0	
Duration of Graves’ disease	54.0 ± 44.6	NA
Treatment of Graves’ disease		NA
Anti-thyroid drug, n	10	
Radioiodine therapy, n	1	
Thyroidectomy, n	1	

The OF cell cultures were performed according to methods described previously [3,4,16,17]. Orbital fat biopsies taken at surgery were minced and placed directly in plastic culture dishes. The cells were incubated in Dulbecco’s modified Eagle’s medium (DMEM) containing 20% fetal bovine serum (FBS) and 5% penicillin–streptomycin in a humidified 5% CO_2_ incubator at 37°C. After the OFs had grown out from the explants, monolayers were passaged serially by gentle treatment with trypsin/ethylenediaminetetraacetic acid (EDTA), and cells were incubated in DMEM with 10% FBS and antibiotics. Cells were stored in liquid N_2_ until needed and used between the third and seventh passage. All reagents were purchased from GenDEPOT (GenDEPOT, Katy, TX, USA)

### Quantitative real-time polymerase chain reaction (qRT-PCR)

For qRT-PCR analysis, the total RNA and miRNA of each orbital fat/connective tissue sample obtained from GO patients and non-GO normal controls were isolated using a TRIzol (Tri Reagent Solution; #AM9738; Ambion) and/or a miRNA isolation kit (MirVana miRNA Isolation Kit; #AM1560; Invitrogen) and then reverse-transcribed into complementary DNA using a cDNA reverse transcription kit (High Capacity cDNA Reverse Transcription Kit; #4368814; Applied Biosystems, Waltham, MA, USA) and a miR reverse transcription kit (TaqMan MicroRNA Reverse Transcription Kit; #4366596; Applied Biosystems). The resulting cDNA was amplified using a thermocycler (ABI Step One Plus Real Time PCR; Applied Biosystems) with a PowerUp SYBR Green Master Mix (#A25918; ABI) and universal PCR master mix (TaqMan No AmpErase UNG; #4324018; ABI). All PCRs were performed according to the manufacture’s recommended method with the RNU6B as an internal control. The catalogue number of the primers used was 002623 for miR-155. The PCR amplification was performed using specific primer pairs as follows:

Interleukin (IL)-6:

        F: 5’- AGACAGCCACTCACCTCTTCAG -3’
        R: 5’- TTCTGCCAGTGCCTCTTTGCTG -3’

Cyclooxygenase (COX)-2:

        F:5’- CCCTTGGGTGTCAAAGGTAA -3’
        R:5’- GCCCTCGCTTATGATCTGTC -3’

Intercellular adhesion molecule (ICAM)-1:

        F:5’- GCCACTTCTTCTGTAAGTCTGTGGG -3’
        R:5’- CTACCGGCCCTGGGACG -3’

ITK:

        F:5’- CACGGCTGCCTGTCAGATTA -3’
        R:5’- TGTGGATGACACATGCCTCT -3’

IL-17:

        F:5’- TCAACCCGATTGTCCACCAT -3’
        R:5’- GAGTTTAGTCCGAAATGAGGCTG -3’

All PCRs were performed in duplicate and normalized to GAPDH expression. The results (fold changes of the threshold cycle [Ct] relative to the control group) were obtained using the 2−ΔΔCt method.

### Transfection with miR-155 mimics and inhibitors

OFs were transfected with miR-155 mimics, inhibitors, and each control according to the respective manufacturer protocols. OF cells (1.5 × 10^6^ OF cells) were seeded in 100-mm plates 24 hours prior to transfection. Media were replaced with antibiotic-free media at the time of transfection. Cells were transfected with the indicated miR-155 mimics (#4464067; Ambion, Austin, TX, USA), inhibitors (#4464085, Ambion), and siITK (#AM16708, Ambion) at concentrations of 100 nM using commercial reagents (Lipofectamine 2000; Invitrogen, Carlsbad, CA, USA) for 6 hours. Cells were cultured for an additional 18 to 42 hours with antibiotic-containing media.

To investigate the effect of miR-155 mimics and inhibitors on IL-1β-induced IL-6, COX2, and ICAM expression, 5 ng/mL of IL-1β was pre-treated for 16h. Then, 24 and 48 hours after further transfection, cells were washed twice with ice-cold phosphate buffered saline (PBS), miRNA and protein of IL-6, COX2, and ICAM, respectively, collected by scraping.

### Cell stimulation

To determine whether overexpression of miR-155 is induced by inflammatory stimulation, OFs from GO and non-GO patients were treated with IL-1β (#100-167AF; Shenandoah, Warminster, PA, USA) and TNF-α (#ab259410; Abcam, Cambridge, UK). Cells were stimulated in DMEM:F12 with 10 ng/mL of IL-1β and TNF-α for the indicated time (0, 3, 6, 16, and 24 hours). Cells were also stimulated in DMEM:F12 with 0, 1, 5, 10, and 20 ng/mL IL-1β and TNF-α for 16 hours. The indicated times and concentrations at each point were determined according to previous publications on GO using primary cultured orbital fibroblasts [2,18,19].

To assess the molecular mechanism of IL-1β–induced miR155 expression, the effects of inhibitors of MEK-1/2 (PD98059; Sigma-Aldrich Corp., St. Louis, MO, USA) (U0126;#sc-222395; Santacruz, TX, USA), NF-κB pathway (SC-514;Calbiochem, La Jolla, CA, USA), c-Jun N-terminal Kinase (JNK)-1/2 (SP600125;Sigma-Aldrich Corp), phosphatidylinositol 3-kinase (PI3-K) (LY294002; Sigma-Aldrich Corp.), p38 mitogen activated protein kinase (MAPK) (SB203580; Sigma Aldrich Corp.), and mTOR (rapamycin) were investigated using GO OFs. Following 1-hour pre-treatment with 20 μM of inhibitors, OFs were stimulated with IL-1b (5 ng/mL) and the expression of miR-155 was determined at 16 hours.

To investigate the effects of ITK inhibitors (BMS509744;CAS 439575-02-7; Tocris Bioscience, Bristol, UK). and Bruton’s tyrosine kinase (BTK)/ITK dual inhibitors (ibrutinib;#PCI-32765; Selleck Chemicals, Houston, TX, USA) on miR-155 expression in GO and non-GO OFs, cells were stimulated with 1μM of BMS and ibrutinib for the indicated time (0, 6, 16, and 24 hours).

### Western blot

The effects of miR-155 mimics on IL-1β-induced IL-6, COX-2, and ICAM-1 release in GO OFs were analysed by Western blotting. Transfected cells were washed with ice-cold PBS, and whole-cell lysates were obtained by incubation on ice for 30 minutes in a cell lysis buffer (RIPA II cell lysis buffer; #R4200-010; GenDEPOT). Lysates were centrifuged at 12,000 × g for 10 minutes, and the cell homogenate fractions were stored at −70°C until use. Protein concentrations were determined by bicinchoninic acid protein assay. Equal amounts of protein (20 μg) were boiled in a sample buffer and resolved by 10% (wt/vol) sodium dodecyl sulfate polyacrylamide gel electrophoresis. Proteins were transferred onto polyvinylidene difluoride membranes (Immobilon; Millipore, Billerica, MA, USA). The samples were probed overnight with primary antibodies (IL-6, #12153; Cell Signaling; COX-2, #ab62331; ICAM-1, #ab53013; IL-1β, #ab9722; TNF-α, #ab6671, Abcam; IL-17, #sc-374218; Santa Cruz Biotechnology, Santa Cruz, CA, USA) in Tris-buffered saline containing Tween 20 (TBST) and 5% skim milk, and washed three times with TBST. Immunoreactive bands were detected with horseradish peroxidase-conjugated secondary antibody and developed using an enhanced chemiluminescence kit (ECL solution; GenDEPOT). The immunoreactive bands were quantified by densitometry and normalized relative to the β-actin level in the same sample.

### Enzyme-linked immunosorbent assay (ELISA)

The effects of miR-155 mimics and inhibitors on ITK release and expression were analysed using a human cytokine ELISA kit (ITK ELISA kit; #MBS284985; MyBioSource, Inc., San Diego, CA, USA) according to the manufacturer’s protocol for three GO and three non-GO OFs from different individuals. Transfected cells were plated into six-well plates and left to adhere overnight. Supernatants were removed at 24 hours, and ITK levels were determined by ELISA.

### Cell viability assays

OF cells were seeded in a 96-well plate with 1.7 × 10^4^ cells per well and treated as follows: fresh culture medium alone (control), fresh culture medium with different concentrations (0, 0.5, 1, 1.5, 10, 20, 25 μM) of ibrutinib, and BMS 509744. Cell viability was assessed by a Cell Counting Kit-8 (CCK-8; Abcam) according to the manufacturer’s instructions. Briefly, after treatment, the CCK-8 solution was added to the culture medium and incubated at 37°C for 4 hours. Optical density was read on a microplate reader (Molecular Devices, Sunnyvale, CA, USA) at an absorbance value of 460 nm. Cell viability was calculated as follows: (experimental group absorbance value / control group absorbance value) × 100%.

### Immunocytochemistry staining (ICC)

For ICC staining, OFs were prepared on coverslips with polyethylene, for 1 h at room temperature. After drying completely, coverslips were sterilized using UV light for 4 h. After the coverslips had been well-rinsed with PBS, the cells were incubated in 100% methanol (chilled to -20°C) at room temperature for 5 min. The cells were then incubated with phosphate-buffered saline (PBS) containing 0.1% Triton X-100 for 10 min, to improve penetration of the antibody. After blocking, the samples were incubated with primary antibodies, including vimentin (1:1,000, #ab92547; Abcam, Cambridge, UK) and α-smooth muscle actin (SMA) (1:1,000, #ab5694; Abcam), in 1% bovine serum albumin (BSA) in PBST for 1 h at room temperature, or overnight at 4°C. The solution was then decanted, and the cells were washed three times in PBS (5 min for each wash). The cells were then incubated with the secondary antibody in 1% BSA for 1 h at room temperature in the dark. The secondary antibody solution was decanted and then washed with PBS three times (5 min per wash). For nuclear counter-staining, FluoroShield™ with 4’,6-diamidino-2-phenylindole dihydrochloride (DAPI; Sigma-Aldrich) was used. DA was performed using Hoechst 33258 (2.5 μg/mL, H1399; Thermo Fisher Scientific, Waltham, MA, USA) in the dark for 15 min at room temperature. Images were obtained using confocal fluorescence microscopy (LSM710; Carl Zeiss, Oberkochen, Germany). Alexa Fluor-594 anti-rabbit (1:10,000, A-11001; Thermo Fisher Scientific) antibodies were used as the secondary antibodies.

### Data analysis

All experiments were performed at least three lines OFs isolated from various patients with GO or non-GO samples. The miRNA and protein levels were measured and normalized in at least three samples harvested from different individuals, and then calculated mean and standard deviation values. Differences between groups were assessed using both independent *t*-tests and One-way ANOVA tests using SPSS V.26.0 (IBM Corporation). In all analyses, p < 0.05 was considered to reflect statistical significance. Three separate experiments were performed using cells from three individuals. Each experiment was repeated three times. Data are expressed as mean ± standard deviation.

## Results

### miR-155 is overexpressed in the orbital tissue of patients with GO and is induced by pro-inflammatory cytokines in OFs

**Fig 1:**
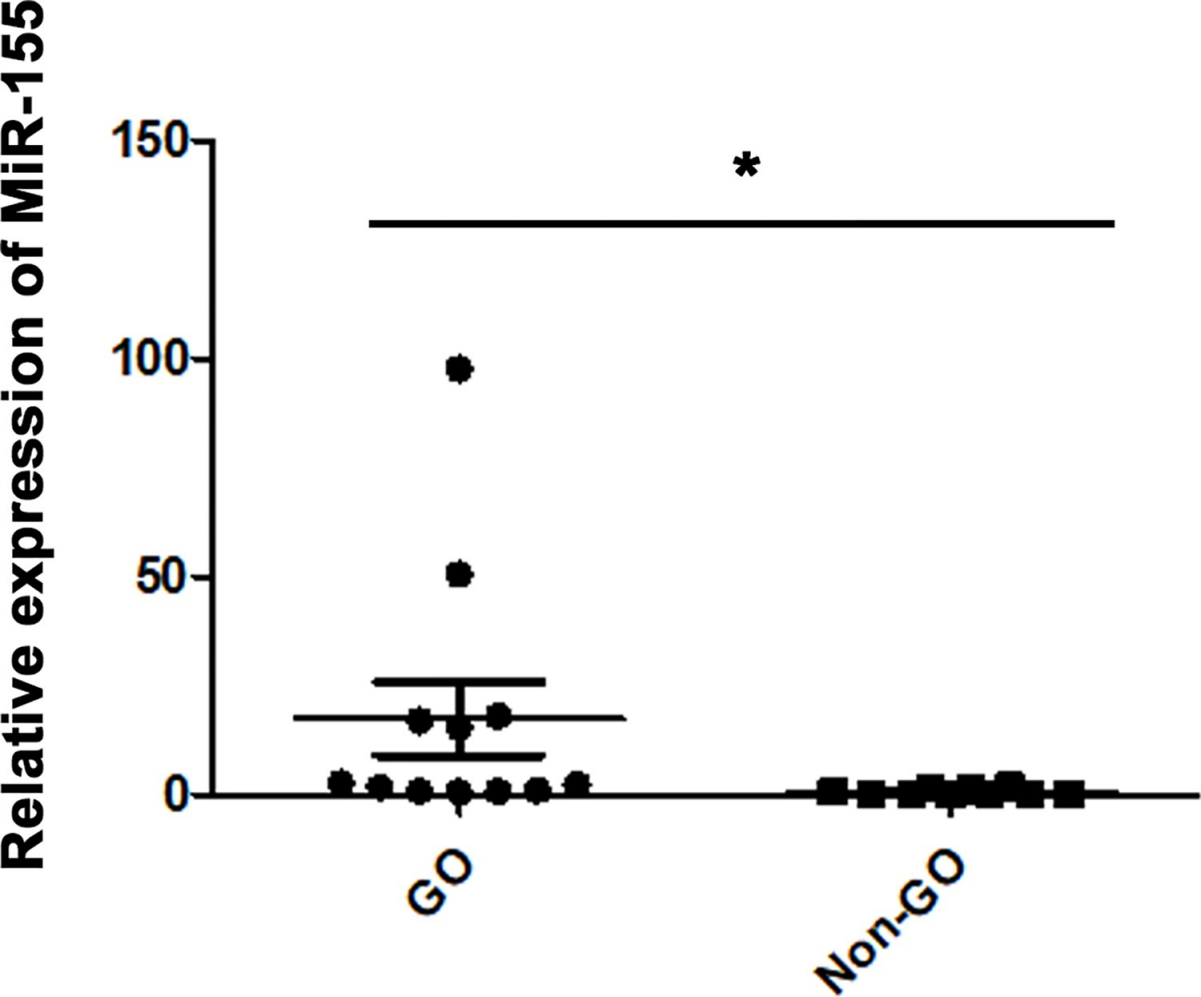
Expression of microRNA (miR)-155 in the orbital tissue of patients with Graves’ orbitopathy (GO, n = 12) and non-GO patients (n = 10). (*P < 0.05, comparison between GO and non-GO in orbital adipose tissues).

To identify whether miR-155 is involved in GO pathogenesis, miR-155 expression levels were determined in orbital fat tissue obtained from 12 GO and 10 non-GO participants using qRT-PCR. Table 1 provides the demographic data. The mean age of all patients with GO was 48.8 ± 15.0 years and eight of them (66.7%) were women. The mean age of the control group was 50.8 ± 12.8 years and there were seven (70%) women. The expression levels of miR-155 were elevated significantly in GO orbital adipose tissues compared with non-GO tissues (p = 0.048; independent *t*-test; Fig 1).

**Fig 2:**
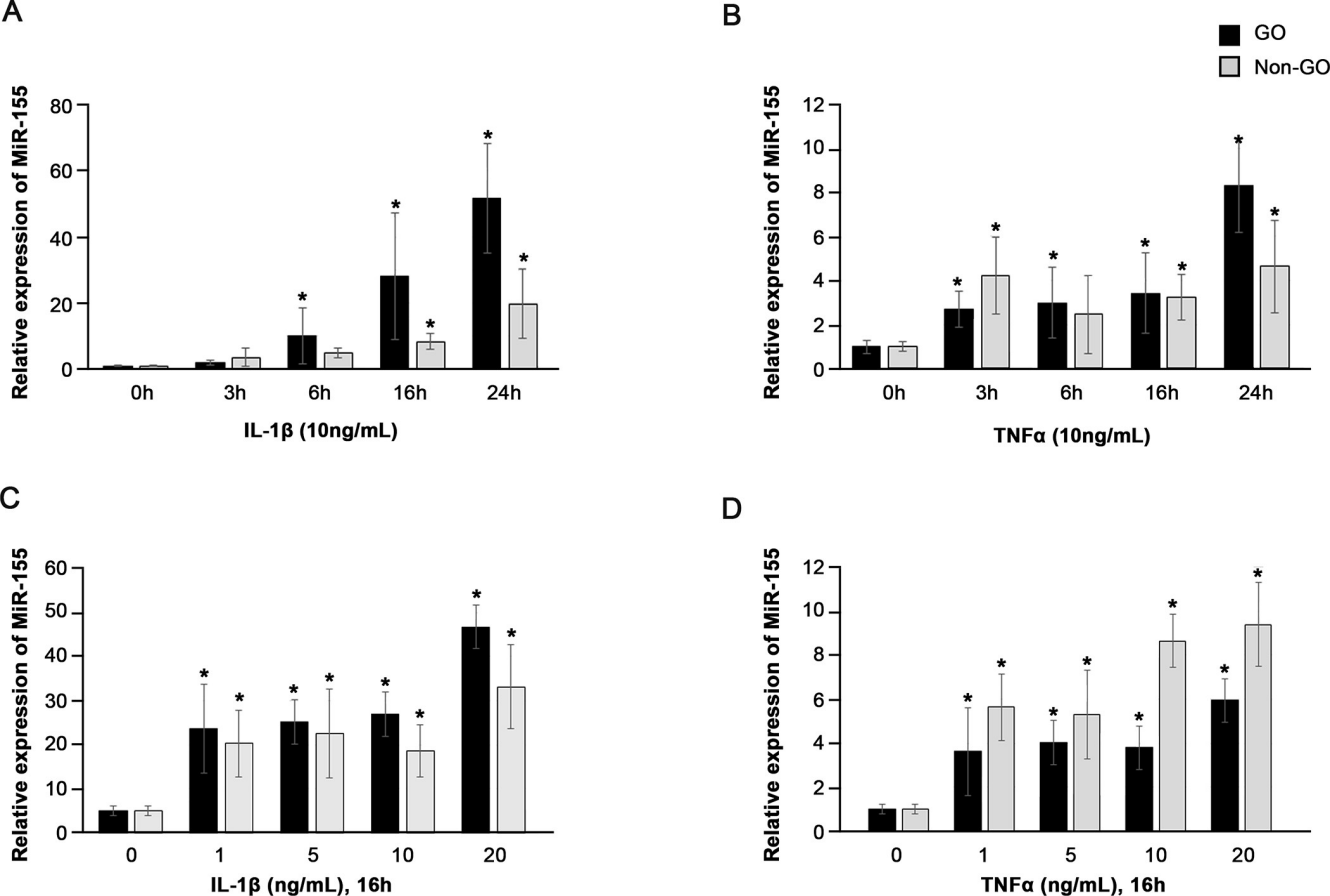
Effects of interleukin (IL)-1β and tumour necrosis factor-alpha (TNF-α) on miR-155 expression in GO (n = 5) and non-GO orbital fibroblasts (n = 5) (OFs). Note that IL-1β and TNF-α tend to induce an increase in miR-155 expression. (A, B) Time-dependent effects of IL-1β and TNF-α, (C, D) Dose dependent effects of IL-1β and TNF-α. Differences between treated and untreated cells are indicated (*P < 0.05, versus time and concentration matched controls).

To determine whether overexpression of miR-155 is induced by inflammatory stimulation, OFs from GO and non-GO patients were treated with IL-1β and TNF-α. OFs from five GO and five non-GO patients were used to measure miR-155 expression following exposure to IL-1β and TNF-α. IL-1β (10 ng/mL) and TNF-α (10 ng/mL) appeared to increase miR-155 expression levels in GO and non-GO OFs (Fig 2A and 2B). IL-1β; moreover, the increase in miR-155 expression in GO OFs occurred in a time-dependent manner (Fig 2A). In the same experiments using OFs from non- GO patients, lower levels of miR-155 were observed in non-GO cells compared with GO OFs (Fig 2A). Although TNF-α (10 ng/mL) showed a tendency to increase miR-155 expression in GO and non-GO OFs, this did not appear to be dependent on the exposure time (Fig 2B). IL-1β (16h) and TNF-α (16h) appeared to increase miR-155 expression levels in GO and non-GO OFs in concentration-dependent manner (Fig 2C and 2D).

### miR-155 suppressed the inflammatory response in GO OFs

**Fig 3:**
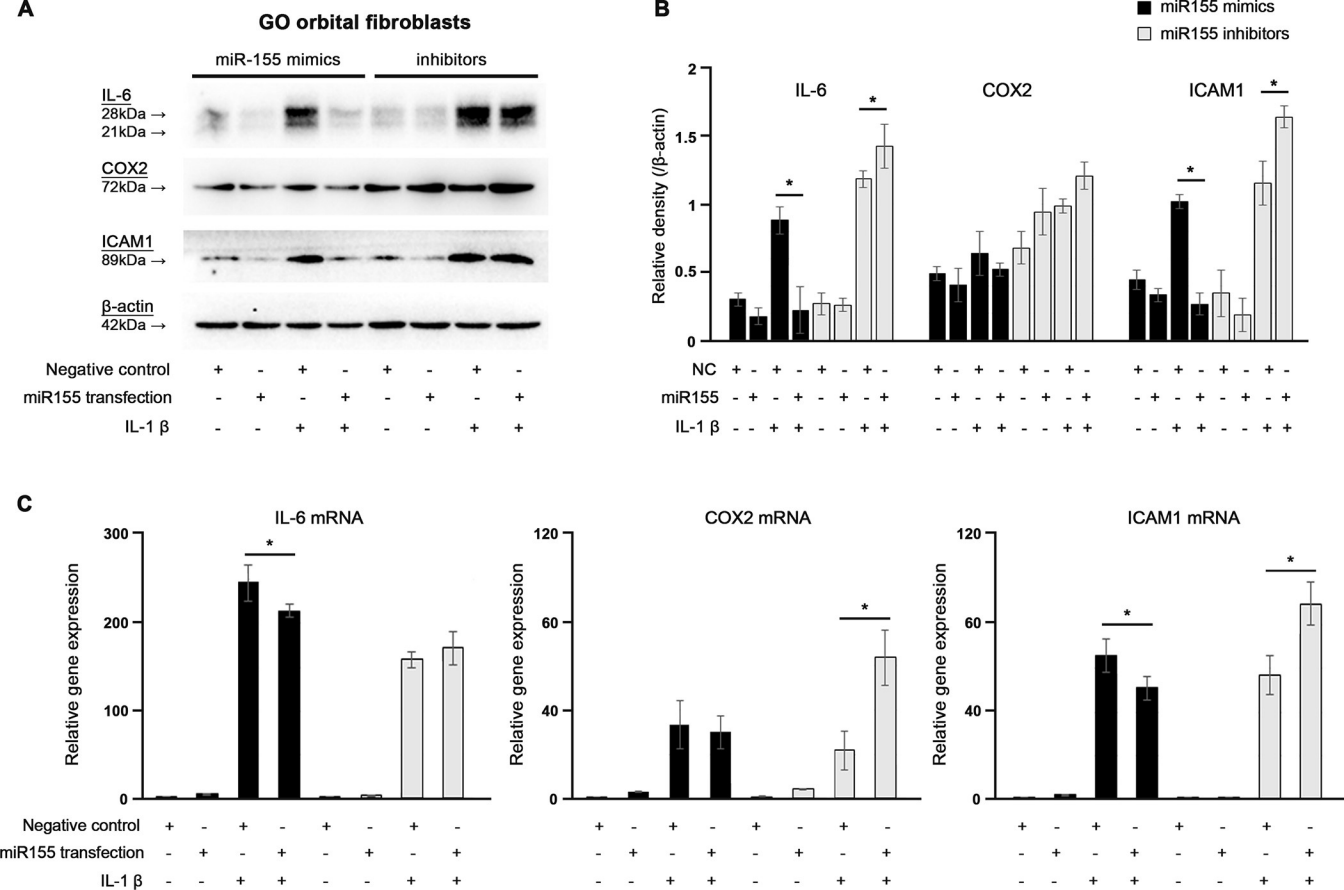
Effects of microRNA-155 mimics and inhibitors on interleukin (IL)-1β induced IL-6, cyclooxygenase (COX)-2, and intercellular adhesion molecule (ICAM)-1 protein and mRNA expression in Graves’ orbitopathy (GO) (n = 4) orbital fibroblasts (OFs). (A, B) IL-1β-induced IL-6 and ICAM1 protein production was reduced by miR-155 mimics, and increased by miR-155 inhibitors, respectively. (*P < 0.05) (C) IL-1β-induced IL-6 and ICAM1 mRNA expression were reduced by miR-155 mimics, and IL-1β-induced COX2 and ICAM1 mRNA expression was increased by miR-155 inhibitors. (*P < 0.05) These experiments were performed at least three times using OFs from at least three GO patients. The results are expressed as the mean ± standard deviation of at least three individual samples, and the graphs are representative of three independent experiments.

To investigate the effect of miR-155 mimics and inhibitors on IL-1β-induced IL-6, COX2, and ICAM expression, we transfected GO OFs with 100 nM miR-155 mimics and inhibitors. In Western blotting analyses, IL-1β-induced IL-6 and ICAM1 protein production was reduced considerably by miR-155 mimics (Fig 3A and 3B).

IL-1β-induced IL-6 and ICAM1 protein production was significantly increased by miR-155 inhibitors (Fig 3A and 3B). The miR-155 mimics and inhibitors did not induce any specific changes in COX2 protein expression (Fig 3A and 3B).

Similar to the results of Western blotting, qRT-PCR showed that IL-1β-induced IL-6 production and ICAM1 mRNA levels were reduced significantly by miR-155 mimics (Fig 3C).

IL-1β-induced COX2 and ICAM1 mRNA expression was increased significantly by miR-155 inhibitors (Fig 3C). The miR-155 mimics did not induce any specific changes in COX2 mRNA expression, and the miR-155 inhibitors did not induce any specific changes in IL-6 mRNA expression (Fig 3C).

### ITK is a functional target of miR-155

**Fig 4:**
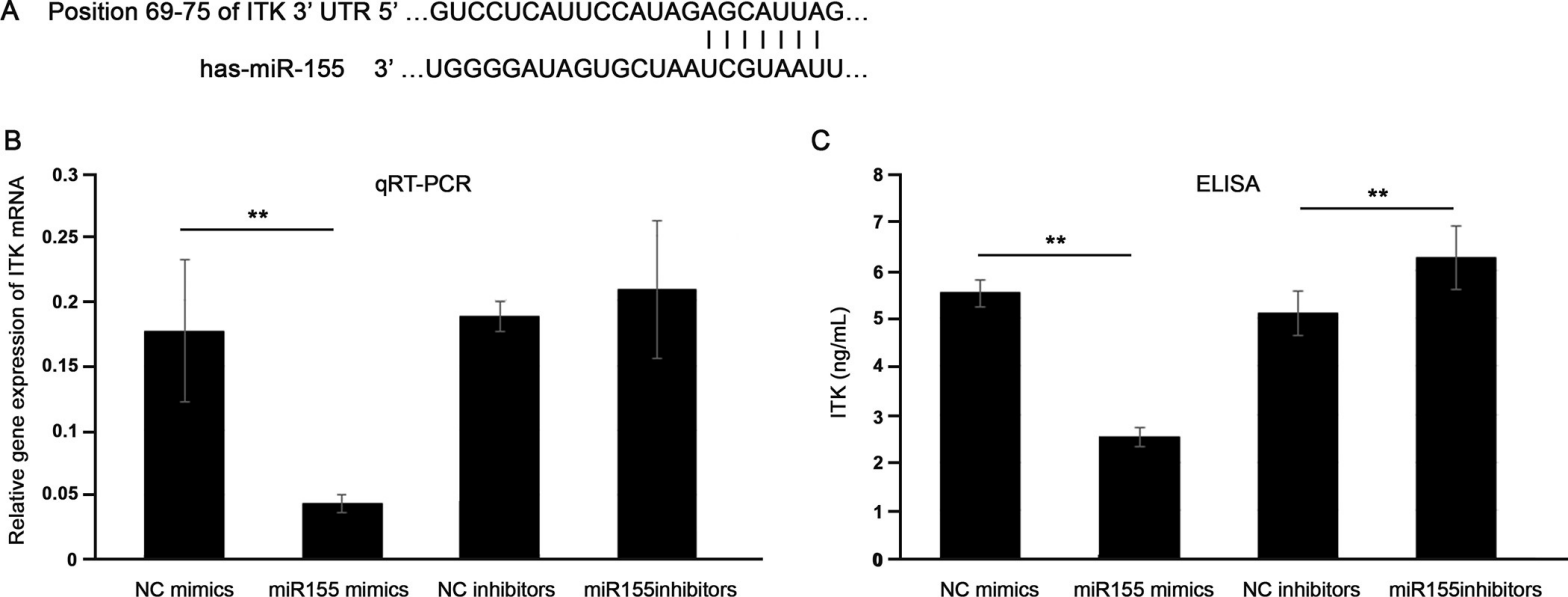
(A) The predicted binding site of microRNA (miR)-155 in the 3’-UTR of interleukin-2-inducible T-cell kinase (ITK) is indicated. (B, C) Effects of miR-155 mimics and inhibitors on ITK mRNA and protein expression in Graves’ orbitopathy (n = 3) orbital fibroblasts. (**P < 0.01) The results are expressed as the mean ± standard deviation of at least three individual samples, and the graphs are representative of three independent experiments.

To elucidate the mechanism by which miR-155 affects the inflammatory response in GO OFs, we performed computational analyses using the online miRNA target database, TargetScan, to identify potential mRNA targets of miR-155. Among the many predicted targets in the miRBase, we focused on ITK as it has a binding site for miR-155 (Fig 4A).

To identify the potential relationship between miR-155 and ITK, qRT-PCR was performed to measure the mRNA levels of ITK in GO OFs with or without miR-155 involvement. The qRT-PCR results indicated that mRNA expression of ITK was downregulated by overexpressed miR-155 via miR-155 mimics (Fig 4B).

Cytoplasmic protein was collected from the GO OFs, which were transfected with miR-control, miR-155 mimic, or miR-155 inhibitor for 24 hours. ELISA revealed that overexpressed miR-155 inhibited ITK protein expression, and downregulation of miR-155 induced an increase in ITK protein expression (Fig 4C).

### ITK is required for inflammatory cytokine expression in OFs

**Fig 5:**
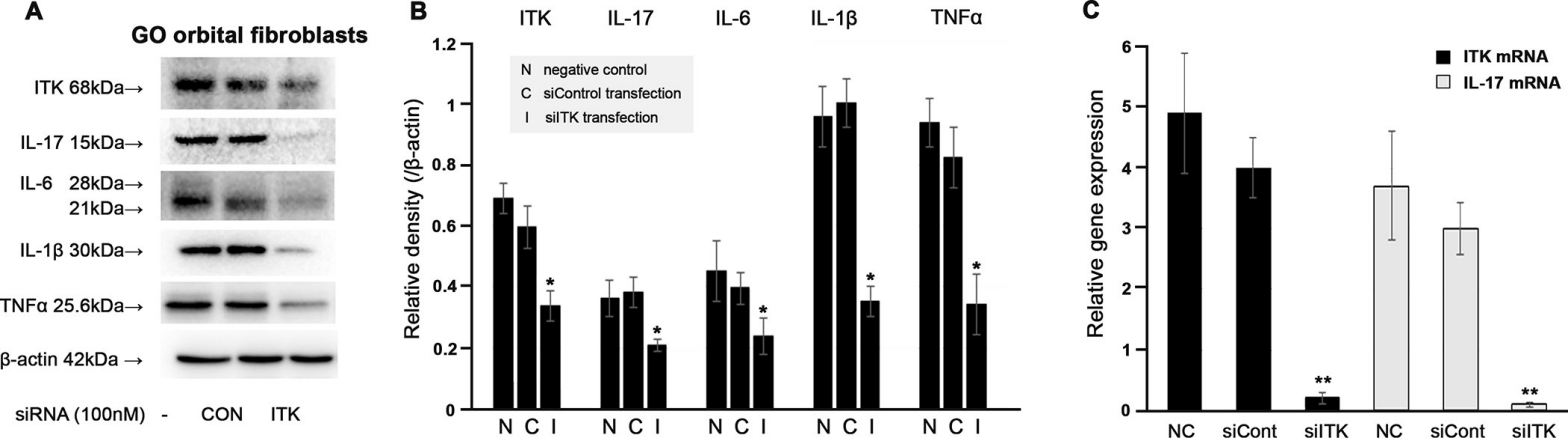
Effects of inhibition of interleukin (IL)-2- inducible T-cell kinase (ITK) on the expression of inflammatory cytokines in Graves’ orbitopathy (GO) (n = 4) orbital fibroblasts (OFs). (A, B) Effects of siITK on the expression levels of ITK, IL-17, IL-6, IL-1β, and tumour necrosis factor-alpha protein, revealed using Western blotting. (*P < 0.05) The results are expressed as the mean ± standard deviation of at least three individual samples, and the graphs are representative of three independent experiments. (C) Quantitative real-time polymerase chain reaction, showed that the mRNA expression of IL-17 was suppressed by siITK treatment. (**P < 0.01) These experiments were performed at least three times using OFs from at least three GO individuals.

To examine whether ITK affects the expression of inflammatory cytokines in GO OFs, GO OFs were transfected with siITK (100 ng/mL, 24 hours) following the manufacturer’s instructions. Knockdown of ITK via siITK transfection induced a decrease in the expression of ITK, IL-17, IL-6, IL-1β, and TNF-α protein (Fig 5A and 5B).

ITK was previously identified as an IL-17 3’-UTR-interacting protein by luciferase assay and mRNA stability assays after ITK overexpression or ITK knockdown [20,21]. Therefore, we further investigated ITK and IL-17 mRNA expression after transfection of siITK. Knockdown of ITK induced a decrease in mRNA expression of ITK and IL-17 (Fig 5C).

### Overexpression of miR-155 by IL-1β is suppressed by NF-κB/JNK/MAPK inhibitors

**Fig 6:**
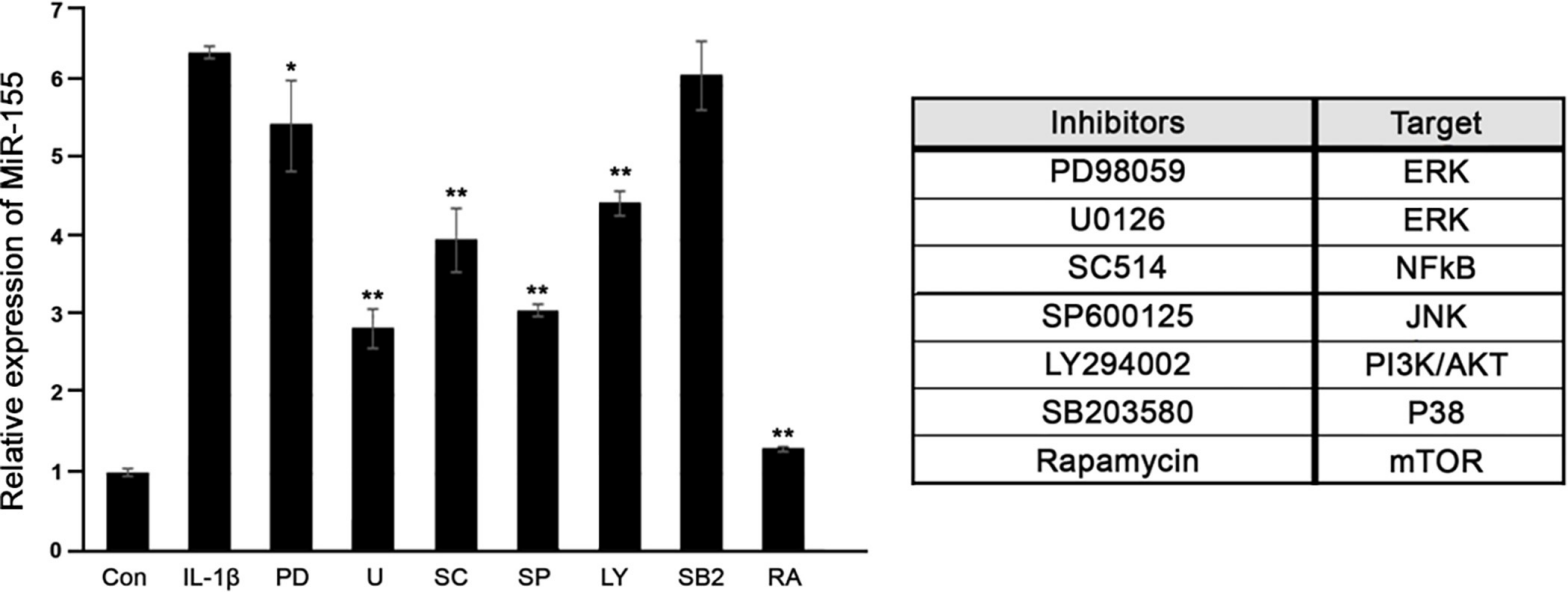
Effects of inhibitors of extracellular signal-regulated kinase, nuclear factor kappa-light-chain-enhancer of activated B cell, c-Jun N-terminal kinase, phosphatidylinositol 3-kinase/Akt, P38 mitogen activated protein kinase, and mammalian target of rapamycin on microRNA-155 expression in Graves’ orbitopathy (n = 3) orbital fibroblasts. (*P < 0.05, **P < 0.01).

To investigate the molecular mechanism underlying IL-1β-induced miR-155 expression, the effects of inhibitors of extracellular signal-regulated kinase, NF-κB, JNK, PI3-K/Akt, P38 MAPK, and mammalian target of rapamycin inhibitors were analysed using GO OFs. IL-1β (5 ng/mL, 16 hours) induced an increase in miR-155 expression; this increase was significantly inhibited by most of the inhibitors, except for p38 MAPK (Fig 6).

### miR-155 expression is suppressed by ITK and BTK/ITK dual inhibitors

**Fig 7:**
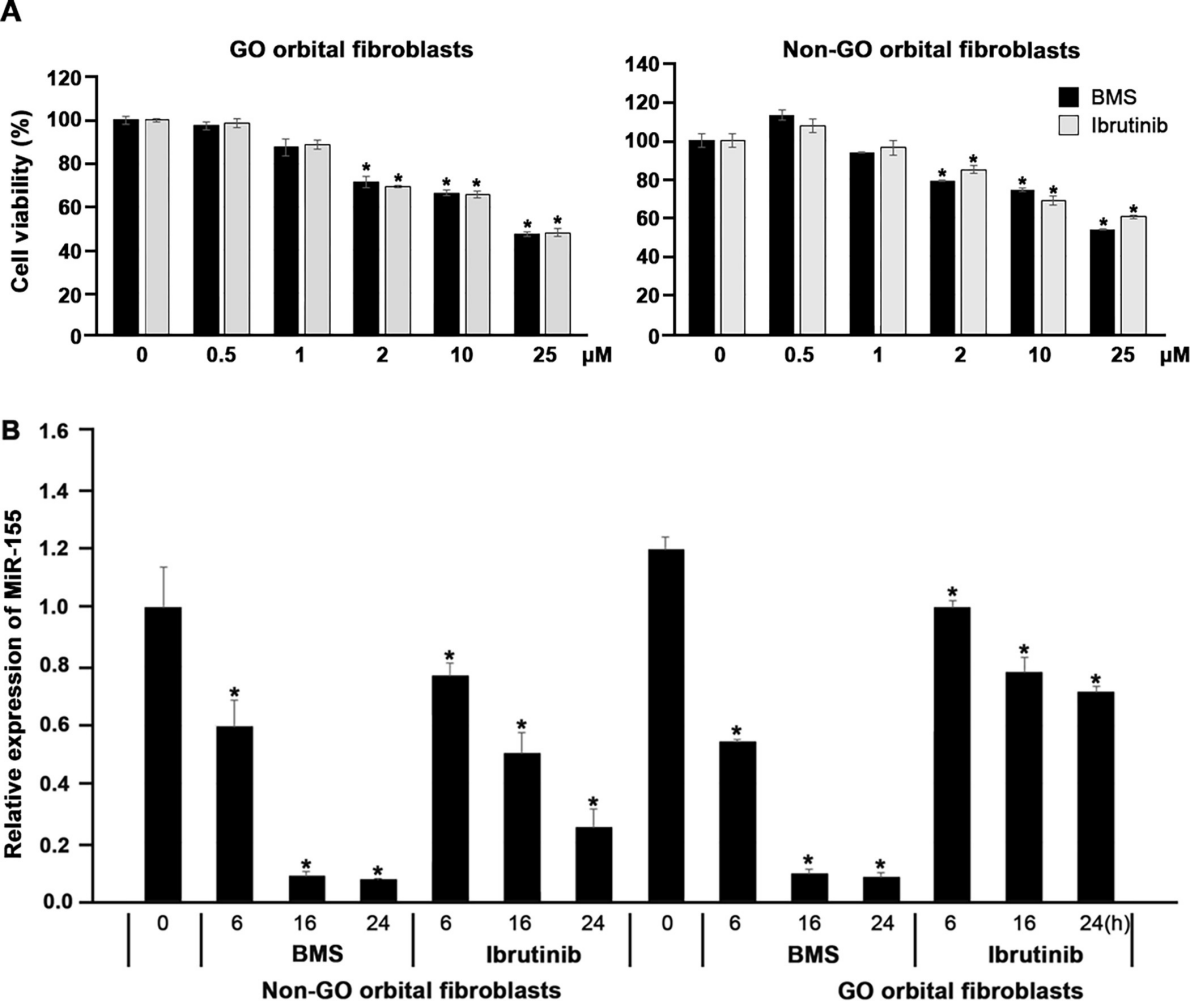
Effect of interleukin-2-inducible T-cell kinase (ITK) inhibitors and Bruton’s tyrosine kinase (BTK)/ITK dual inhibitors on the viability and microRNA-155 expression of orbital fibroblasts in Graves’ orbitopathy (GO) (n = 3) and non-GO (n = 3). (A) Based on the MTT results, exposure of cells to ≤ 1 μM of ITK inhibitors and BTK/ITK dual inhibitors for 24 hours did not reduce cell viability to levels below 80% in GO OFs or 90% in non-GO OFs. (B) The expression of miR-155 was significantly downregulated by treatment with ITK inhibitors and BTK/ITK dual inhibitors, in a time-dependent manner in both GO and non-GO OFs. Differences between treated and untreated cells are indicated (in contrast to cells no treated with ITK, BTK/ITK inhibitors, *P < 0.05).

To evaluate the effect of ITK inhibitors and BTK/ITK dual inhibitors on cell viability, OFs were seeded onto 24-well culture plates (1 × 10^5^ cells per well) and treated with different concentrations of ITK inhibitors and BTK/ITK dual inhibitors for 24 hours. To determine the nontoxic concentrations of ITK inhibitors and BTK/ITK dual inhibitors in GO and non-GO OFs, an MTT (3-[4,5-dimethylthiazol-2-yl]-2,5 diphenyl tetrazolium bromide) assay was performed. Exposure of cells to ≤ 1 μM of ITK inhibitors and BTK/ITK dual inhibitors for 24 hours did not reduce cell viability to levels below 80% in GO OFs and 90% in non-GO OFs (Fig 7A). Thus, in this study, 1 μM of ITK inhibitors and BTK/ITK dual inhibitors were applied for 24 hours.

The effects of ITK inhibitors and BTK/ITK dual inhibitors on miR-155 expression in GO and non-GO OFs were investigated. The expression of miR-155 was significantly downregulated by treatment with ITK inhibitors and BTK/ITK dual inhibitors in a time-dependent manner in both GO and non-GO OFs (Fig 7B).

## Discussion

In the present study, we investigated the role of miR-155 in the regulation of inflammation in an in-vitro model of GO. We compared the expression levels of miR-155 between GO and non-GO orbital adipose tissue. The expression of miR-155 was significantly higher in orbital adipose tissues from GO than non-GO patients. MiR-155 expression was upregulated by inflammatory stress in GO and non-GO OFs. IL-1β-induced IL-6 and ICAM1 protein production was significantly decreased and increased by miR-155 mimics and inhibitors, respectively. These results indicate that miR-155 plays a specific role in the regulation of inflammation in OFs and may act as an anti-inflammatory factor. Indeed, we previously investigated the role of miR-146a in the inflammation in GO OFs [2]. Similar to miR-155, miR-146a expression was upregulated by IL-1β and was significantly higher in GO orbital tissue than non-GO orbital tissue, indicating that miR-146a has a positive effect on the anti-inflammatory process.

Specific miRNAs, such as miR-155 and miR-146a, have been linked to the inflammatory response [22]. Indeed, both of those miRNAs are relatively well-known to play a role in inflammatory autoimmune disease [23]. One interesting previous report proposed the hypothesis that miR-155 and miR-146a may have opposite impacts on inflammatory responses in the pathogenesis of GO; however, the authors did not experimentally confirm this premise [24]. This hypothesis, however, is supported by the results from another study, which demonstrated that miR-155 promotes inflammation by targeting SOCS1 and SHIP1, which are negative regulators in the TLR4/NF-κB pathway [13]. In contrast to miR-155, miR-146a inhibits inflammation by suppressing TNF receptor-associated factor 6 and IL-1 receptor-associated kinase. However, in the present study, we found that miR-155 had anti-inflammatory effects in GO OFs, similar to those of miR-146a. Given that miRNAs have multiple mRNA targets and exhibit different responses in different cell lines, the results were unexpected. In a recent report [25], the expression levels of miR-155 and miR-146a increased more than five-fold in kidney samples from diabetic nephropathy (DN) patients compared with the controls. During the induction and progression of DN, a chronic low-grade inflammation disorder [26], miR-155 and miR-146a expression increased gradually [25].

Similarly, there have been several reports that the expression of miR-155 increases in diseased tissue [12,25,27–29]. Li *et al* [12] reported that miR-155 is overexpressed in the serum and atherosclerotic lesions of atherosclerosis patients; additionally, miR-155 expression is increased in a dose- and time-dependent manner by inflammatory stress. The authors demonstrated that miR-155 inhibits CARHSP1, which affects the stability of TNF-α mRNA, concluding that increased miR-155 levels relieve inflammation and play a protective role via miR-155-CARHSP1-TNF-α pathway signaling. Stanczyk *et al* [29] found that the overexpression of miR-155 induced by inflammatory stress downregulates the production of matrix metalloproteinases (MMPs) 1 and 3, which are important markers of the inflammatory effect of rheumatoid arthritis. The authors concluded that miR-155 might function as a protective miRNA that downregulates local expression of MMPs. Contrary to the previously mentioned reports, however, Murugaiyan *et al* [30] reported that miR155 (-/-) mice had a delayed course and less severe autoimmune encephalomyelitis, and less inflammation in the central nervous system. Furthermore, another study reported a pro-inflammatory role for miR-155 in bone marrow-derived macrophages [31]. Likewise, there are some discrepancies regarding the role of miR-155 in inflammation. We believe that the function of miR-155 is highly dependent on the context and cell types due to its dynamic expression pattern and suppression of multiple target genes [12].

Inhibitors of various signals cascade such as MEK-1/2, JNK, p38 MAP kinase, and PI3-K were used to investigate which signal cascade controls the increase in miR-155 expression by IL-1β in orbital fibroblasts. The results indicated that the increase in miR-155 expression induced by IL-1β was inhibited by MEK-1/2, JNK, and PI3-K inhibitors, except p38 inhibitors. Although we are not able to explain the exact mechanism regard to this result, we assumed that the link between miR-155 and MAP kinase might affect the result. P38 is a component of the MAPK signaling pathway and MAPK13 and MAPK14 are known to be potential target genes of miR-155 [32]. Furthermore, miR-155 mimics decreased the expression of p38 compared to control group, whereas the inhibitors increased the levels of p38 in endometrial cell line [33].

In the present study, we investigated the potential relationship between miR-155 and ITK. We found that miR-155 inhibits ITK, an important mediator of inflammation. ITK is involved in numerous signaling pathways and is an important regulator of various signaling pathways in immune cells that contribute to the development of numerous inflammatory diseases [34]. Although ITK is a component of the T-cell receptor signaling pathway [35], further studies are required to clarify the role of ITK in various cell types and signaling pathways, given the numerous methods now available for inhibiting ITK signaling activity and the development of new small-molecule inhibitors by pharmaceutical companies.

ITK regulates the production of Th2, Th9, and Th17 cytokines [15,35–38]. Studies have shown that ITK is involved in the pathogenesis of various inflammatory diseases [39,40]. The loss of ITK or its activity, either by mutation or through the use of inhibitors, has led to beneficial outcomes in experimental models of asthma, inflammatory bowel disease, and multiple sclerosis, among other diseases. In the present study, we examined whether the effects of ITK on the expression of inflammatory cytokines in GO OFs, and the knockdown of ITK via siITK transfection, induced a decrease in the expression levels of ITK, IL-17, IL-6, IL-1β, TNF-α, and IL-17. These findings indicate that ITK should be a target for drug development. Ibrutinib, a small-molecule BTK inhibitor proven to be clinically effective in various B-cell lymphoproliferative diseases, also inhibits ITK. BMS509744 is a selective ITK inhibitor. In the present study, we used ibrutinib and BMS as ITK inhibitors; the expression of miR-155 was significantly downregulated by treatment with ITK inhibitors and BTK/ITK dual inhibitors in a time-dependent manner, in both GO and non-GO OFs. TEC kinases, recognized as important regulators of the signaling cascade in immune cells, include BTK, resting lymphocyte kinase/T-cell X chromosome kinase, bone marrow expressed kinase, and ITK [41].

In conclusion, our results showed a potential relationship between miR-155 and ITK in the context of GO OFs. Overexpression of miR-155 repressed ITK expression and relieved inflammation. Thus, we found that miR-155 has anti-inflammatory effects in GO OFs. This discovery could lead to new therapeutic approaches to the treatment of GO.

## Supporting information

S1 Fig Confocal microscope images showing the expression of vimentin (red) and live cell nuclei (DAPI; blue) in orbital fibroblasts (OFs) (A, C, E). The presence of vimentin in primary cultured OFs from Graves’ orbitopathy (GO) patients. α-SMA immunostaining was not found in primary cultured OFs from GO patients (B, D, F). Bars = 10 μm.
(PDF)

S2 Fig Full-length gels of representative Western blots for Figs 3 and 5. (PDF)
