# Supplementary figures and images for: MicroRNA-155 acts as an anti-inflammatory factor in orbital fibroblasts from Graves’ orbitopathy by repressing interleukin-2-inducible T-cell kinase
Source: PLoS One. 2022 Aug 18;17(8):e0270416. doi: 10.1371/journal.pone.0270416 (PMC9387810; doi:10.1371/journal.pone.0270416)

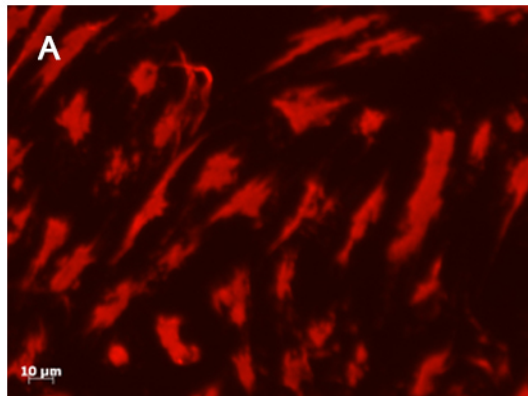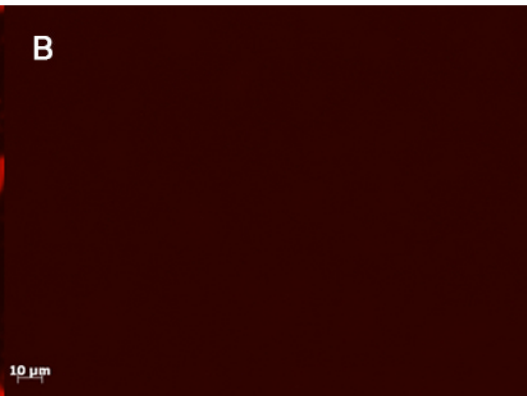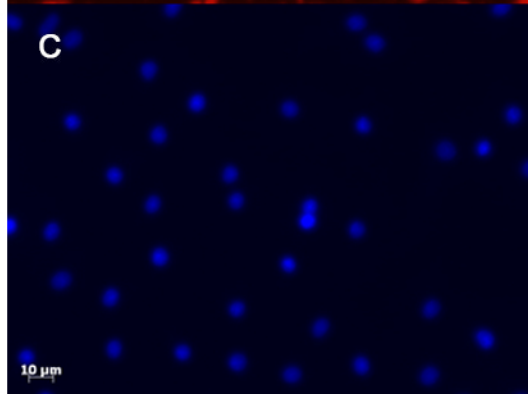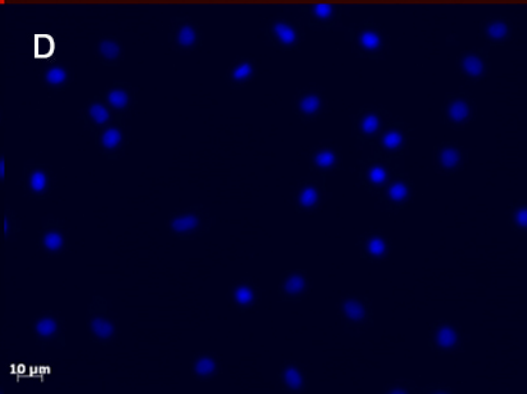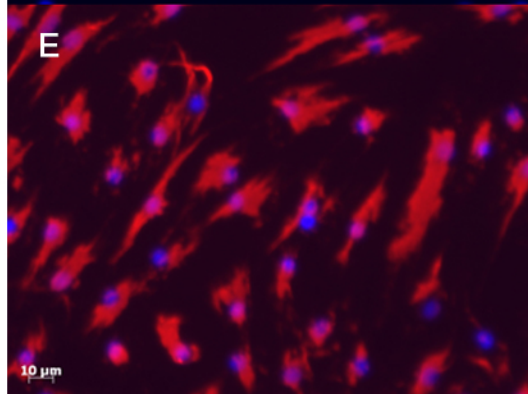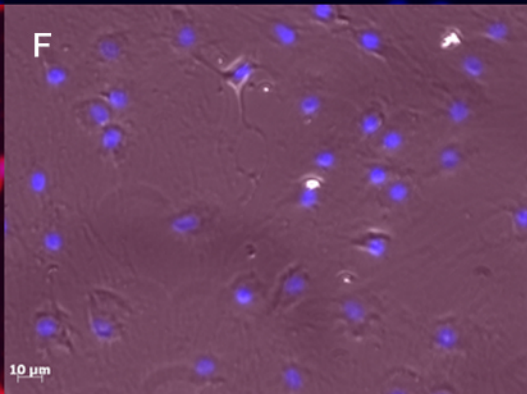

Supplement: S1 Fig — The presence of vimentin in primary cultured OFs from Graves’ orbitopathy (GO) patients. α-SMA immunostaining was not found in primary cultured OFs from GO patients (B, D, F). Bars = 10 μm. (PDF) [file pone.0270416.s001.pdf]

Figure 3.

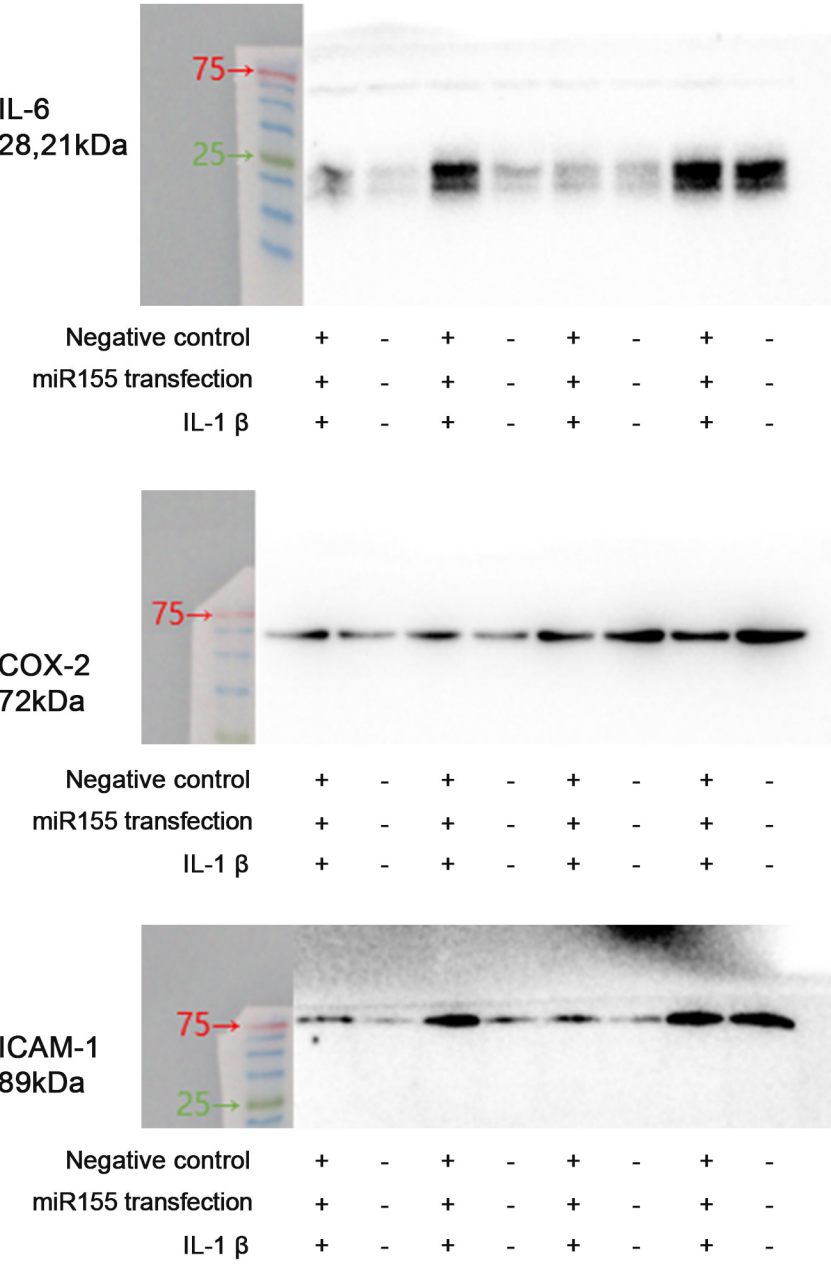

Figure 5.

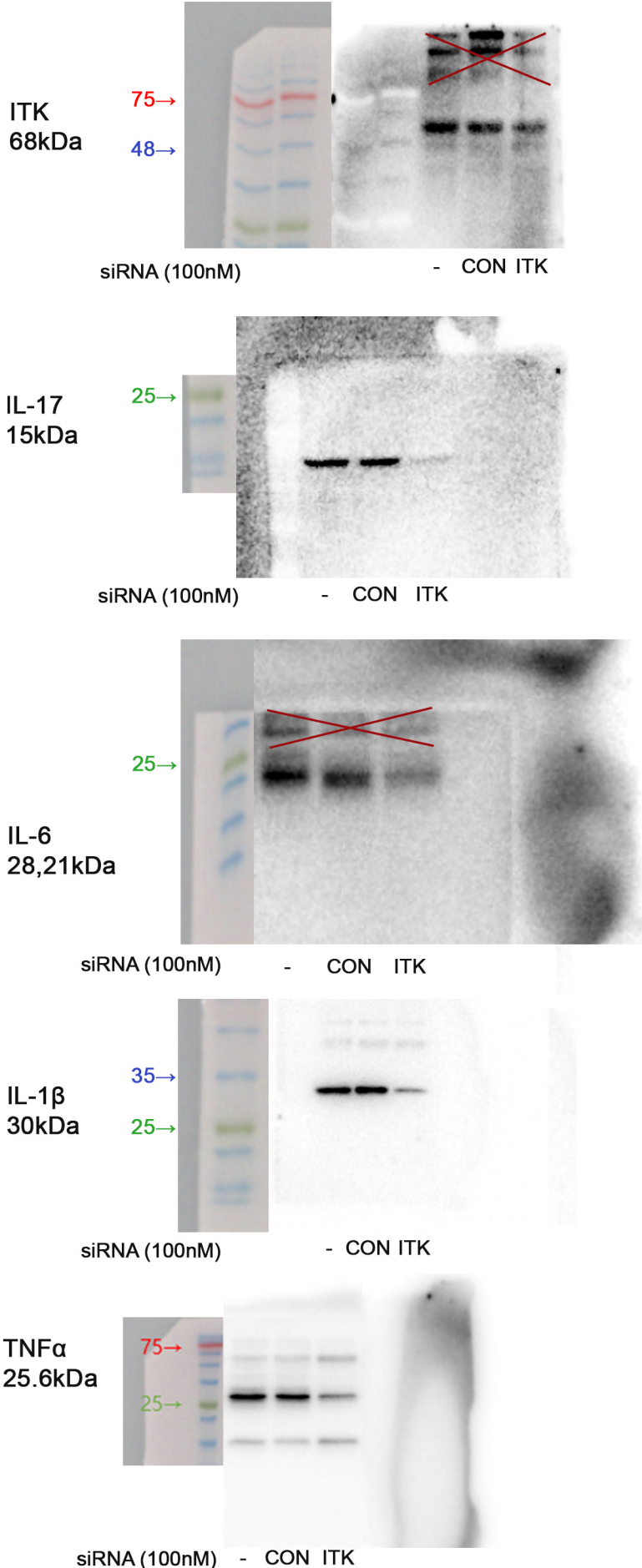

Supplement: S2 Fig — (PDF) [file pone.0270416.s002.pdf]
